# Discovering Novel Bioactivities of Controversial Food Additives by Means of Simple Zebrafish Embryotoxicity (ZET) Assays

**DOI:** 10.3390/toxics11010008

**Published:** 2022-12-22

**Authors:** Dinh Duy-Thanh, Nguyen Bich-Ngoc, François Van den Bossche, Nguyen Lai-Thanh, Marc Muller

**Affiliations:** 1 Laboratory for Organogenesis and Regeneration, GIGA Institute, University of Liège, 4000 Liège, Belgium; 2 LEMA, Urban and Environmental Engineering Department, University of Liège, 4000 Liège, Belgium; 3 Molecular Physiology Research Unit, Faculty of Medicine, University of Namur, 5000 Namur, Belgium; 4 Department of Cell Biology, Faculty of Biology, VNU University of Science, Hanoi 100000, Vietnam

**Keywords:** food additives, bioactivities, zebrafish, embryos, food safety, developmental toxicology

## Abstract

The rising concerns about controversial food additives’ potential hazardous properties require extensive yet animal-minimized testing strategies. Zebrafish embryos are the ideal in vivo model representing both human and environmental health. In this study, we exposed zebrafish embryos to eight controversial food additives. Our results indicate that Sodium Benzoate is a Cat.3 aquatic toxicant, while Quinoline Yellow is a strong teratogen. At high concentrations, non-toxic chemicals induced similar phenotypes, suggesting the impact of ionic strength and the applicability of the darkened yolk phenotype as an indicator of nephrotoxicity. Three food additives showed unpredicted bioactivities on the zebrafish embryos: Brilliant Blue could weaken the embryonic yolk, Quinoline Yellow may interfere with nutrient metabolism, and Azorubine induced precocious zebrafish hatching. In conclusion, the zebrafish embryo is ideal for high throughput chemical safety and toxicity screening, allowing systematic detection of biological effects—especially those unexpected by targeted in vitro and in silico models. Additionally, our data suggest the need to reconsider the safety status of food additives Quinoline Yellow, Brilliant Blue, Sodium Benzoate, and other controversial food additives in further studies, as well as pave the way to further applications based on the newly found properties of Brilliant Blue and Azorubine.

## 1. Introduction

Almost every human eats and drinks a considerable daily amount of food additives (FAs), accumulating towards 3.6–4.5 kg/year [1]. These additives are used to improve the foods’ taste, texture, aesthetic, and shelf life, representing a wide range of different chemicals with various properties. While commonly consumed worldwide, these compounds are increasingly attracting concerns about their potential impacts on human and environmental health.

Scientific and public debates on FAs’ safety arose in the 1970s regarding the alleged neurobehavioral effects of some food additives [2]. In the 2000s, the so-called “Southampton study” again stirred the argument with the demonstration that consumption of FA mixtures may relate to hyperactivity in children [3], leading to long scrutiny of the infamous “Southampton Six” (Tartrazine, Quinoline Yellow, Sunset Yellow, Azorubine, Ponceau 4R, and Allura Red) by both scientists and legislators. Another research, the “Liverpool study”, showed that FA mixes might synergistically affect the viability and differentiation of mice NB2 neuroblastoma cells [4], adding three more FA suspects: Brilliant Blue, Monosodium Glutamate, and Aspartame. Since then, there have been various studies on the potential health effects of food additives, both individually and in mixtures [5,6,7].

The rise in safety and toxicity studies has provided legislators with vast shreds of evidence to frequently and scientifically update their FA policies. However, there is a wide mismatch among policies worldwide on the safety level of each additive, largely due to the different rates of scientific updates, as well as to the different viewpoints of weighing evidence—for instance, the differences in “acceptable daily intakes” (ADIs) issued by the two most notable regulators: the European Food Safety Authority (EFSA) and the Joint FAO/WHO Expert Committee on Food Additives (JECFA) (Supplementary Table S1). Nevertheless, there is always the need for more comprehensive and highly reliable research into the different aspects of FA safety and potential toxicity, and any new piece of evidence on this topic is valuable for policymakers to reevaluate the substances [8].

On the other hand, FAs are also listed among emerging water contaminants in all aquatic systems, from sewage to the ocean [9,10,11]. While multiple efforts are put into removing the pollutants, research into these compounds’ potential impacts on aquatic organisms is also required. Indeed, some of these compounds, such as Carmine and Sucralose, have already been shown to be aquatoxic, threatening global water environments [12,13].

The need for more studies into FAs’ potential effects on both human and environmental health is in line with the One Health concept. It advocates the use of models that can represent both aquatic ecosystems and humans. These models should ideally be in vivo vertebrates, representing the complexity of an entire organism and maximizing the chance to capture unexpected outcomes. However, the recent trend of applying the 3R principle also requires minimizing the use of animals. Therefore, zebrafish embryos are a perfect candidate for this task: Firstly, despite being a complete lifeform, the zebrafish embryos up to the free feeding stage (120 h post-fertilization—hpf) are not legally recognized as animals in the EU [14], thus totally complying with the 3R. Secondly, the zebrafish is an aquatic vertebrate whose genome shares 70% orthologous genes with humans [15], hence representing both environment and human health. Thirdly, the zebrafish’s rapid embryogenesis allows observation and recapitulation of multiple targets and processes occurring during early development, which can be easily observed through the transparent chorion. Additionally, the fish’s high fecundity and low maintenance cost offer the prospects for developing high-throughput assays [16,17,18]. These advantages have made the zebrafish embryotoxicity test (ZET) an increasingly recognized tool in chemical safety screening for both environmental and biomedical applications [18,19,20,21,22].

Over the years, the zebrafish embryotoxicological toolbox has been supplemented with various advanced techniques, such as transgenic reporter lines or automated phenotyping [23,24,25] to increase experimental throughput and simplify training, or-omics tools such as RNA-Seq [22,26] that enable researchers to explore the mechanisms involved in a chemical’s bioactivity. These methods, while screening for chemicals’ toxicity, often mainly focus on preset endpoints, such as lethality, simple morphological defects, or expression of a reporter gene. However, one big advantage of the embryonic zebrafish model is that specific phenotypes induced by a chemical can give hints to the underlying biological process, which is extremely important when it comes to the safety assessment of chemicals. Following up on unexpected phenotypes observed in zebrafish embryos may serve as the starting point for mechanistic studies on toxico-/pharmacology [22,26,27,28].

In this study, we employed the zebrafish embryos as the model system to investigate the potential biological effects of controversial food additives, selected from the “Liverpool” and “Southampton” studies [3,4]. Thereby, we also demonstrate morphological phenotyping as an effective tool in suggesting chemicals’ mode of action involved.

## 2. Materials and Methods

### 2.1. Materials

KCl and NaCl were obtained from Sigma Aldrich (Hoeilaart, Belgium), MgSO_4_ from VWR (Leuven, Belgium), and the phosphate-buffered solution (PBS) was purchased from Life Technologies (Gent, Belgium).

Eight FAs (analytical grade) were purchased either from Sigma Aldrich (Hoeilaart, Belgium) or, for Aspartame, from Alfa Aesar (Lancashire, UK), as listed in Table 1. Stock solutions and serial dilutions for each chemical were appropriately prepared in E3 (5 mM NaCl, 0.17 mM KCl, 0.4 mM CaCl_2_, and 0.16 mM MgSO_4_) for all embryonic tests.

### 2.2. Toxicological Testing Procedure

Zebrafish wildtype strain AB was maintained in a Techniplast rearing system under a 14:10-h light/dark photocycle within the Zebrafish Facility (GIGA-Research, University of Liège). After breeding, eggs were collected into E3 medium. At 3–4 h post-fertilization (hpf), fertilized and healthy embryos were selected and distributed into 6-well plates at 25 embryos/well containing 5 mL of E3 medium supplemented with appropriate concentrations of test compounds, then incubated at 28 °C.

Embryonic mortality and morphology rates were monitored, dead embryos were removed, and solutions were renewed daily until four days post-fertilization (4 dpf). The staging was based on Kimmel et al. [29], and the lethality endpoints were from the OECD’s Fish Embryo Toxicity (FET) test [19]. Photos were taken using an M165 FC stereomicroscope (Leica).

The experiments tested six to nine concentrations chosen following a range-finding test. All experiments were carried out at least in duplicate on n = 50 embryos per test/condition, including control.

### 2.3. Statistical Analysis

The numbers of dead and malformed embryos were subjected to mixed effects logistic regression to take experimental batch effects into account. Estimated mortality and malformation rates at each chemical concentration, together with their 95% prediction intervals, were obtained using the R packages “lme4” [30] and “merTools” [31]. No observed adverse effect concentrations (NOAECs) were also determined from the tested concentrations using “lme4”. While there is a growing call to replace the NOAEC with the NEC (no-effect concentration, obtained by fitting statistical models) [32], we chose the NOAEC in order to compare our results with FAs’ safety legislation, which is still primarily based on the no observed adverse effect level (NOAEL) from animal studies [33,34,35,36,37,38,39,40,41,42,43,44,45].

Other toxicological indices, including median lethal concentrations (LC_50_), median effective concentrations (EC_50_), and the “teratogenic indices” (TI, defined as the ratio between LC_50_ and EC_50_), were obtained by fitting two-parameter log-logistic function with the R package “drc” [46]. Statistical results were then plotted using GraphPad Prism v9 for Windows.

### 2.4. Target and Functional Prediction

For the additives that induced distinct phenotypes in zebrafish embryos, in silico analysis using online tools was performed to obtain a first hint concerning possible biological processes (BP) involved. Two predictive platforms were employed to predict potential protein targets of each FA: the Target Net (http://targetnet.scbdd.com/calcnet/index/) (accessed on 15 December 2022) [47] and the Swiss Target Prediction (http://www.swisstargetprediction.ch) (accessed on 15 December 2022) [48], using the respective websites’ default settings. Due to the differences in algorithms and display of potential protein targets of the two platforms, we defined a potential target hit as having a probability score >0 in any platform.

The protein hits were then subjected to the DAVID database (https://david.ncifcrf.gov) (accessed on 15 December 2022) for the gene ontology (GO) analysis with the EASE score set to 1. All web-based analyses were performed on 26 November 2022.

## 3. Results and Discussion

### 3.1. General Toxicological Results

**Figure 1:**
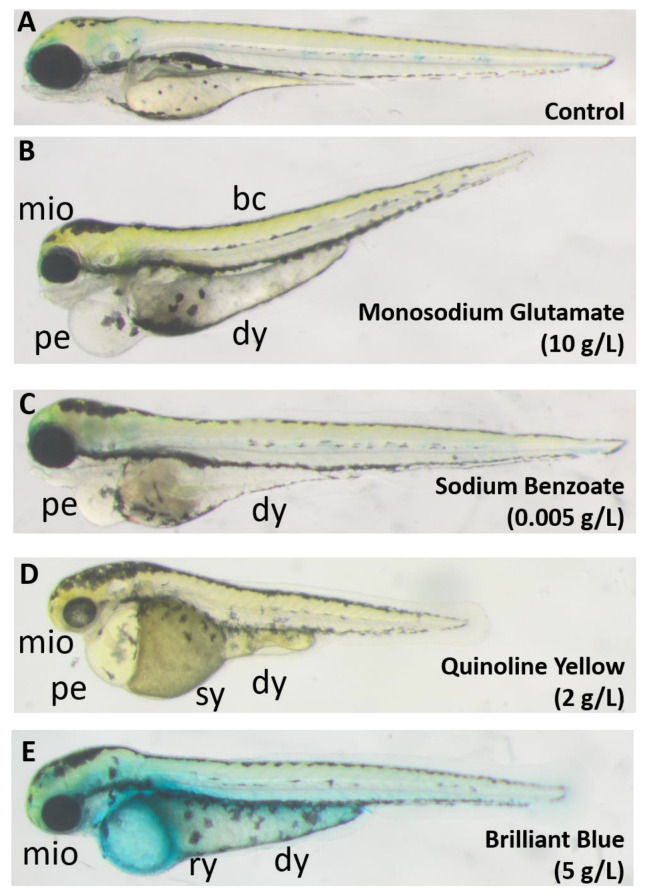
Typical defects following treatment with the test food additives. (**A**) Control; (**B**) Monosodium glutamate; (**C**) Sodium Benzoate; (**D**) Quinoline Yellow; (**E**) Brilliant Blue. Images were taken at 3 dpf. bc: body curvature; dy: darkened yolk; mio: microphthalmia; pe: pericardial edema; sy: swollen yolk; ry: ruptured yolk.

After four days of semi-static (daily medium renewal) exposure, the effects of each substance on zebrafish embryonic morphology and lethality were determined, as well as the corresponding concentration–response relationship. Apart from Aspartame (Asp, E951), which induced no observable effect at all tested concentrations (from 50 mg/L to its saturation at 10 g/L), all other compounds affected zebrafish embryonic development in dose-dependent manners, causing malformations eventually followed by death. The most common defects were pericardial edema, darkened yolk sac, body curvature, and retardation (Figure 1). Among these morphological defects, edema, curvature, and retardation phenotypes are commonly observed in toxicological studies and have been previously linked each to several biological modes of action [28,49]; therefore, they will not be further discussed in this study. On the other hand, an additional defect scarcely mentioned in the literature was the darkened yolk observed for all the compounds except Aspartame, which will be further investigated in the next Section 3.2. Furthermore, three compounds caused small eye (microphthalmia), while two FAs induced substance-specific phenotypes: Quinoline Yellow (QY, E104) caused swollen yolk, and Brilliant Blue (BB, E133) induced yolk rupture (Figure 1D,E) in 20% or more of surviving larvae. These two phenomena will also be followed-up in Section 3.3 and Section 3.4.

**Figure 2:**
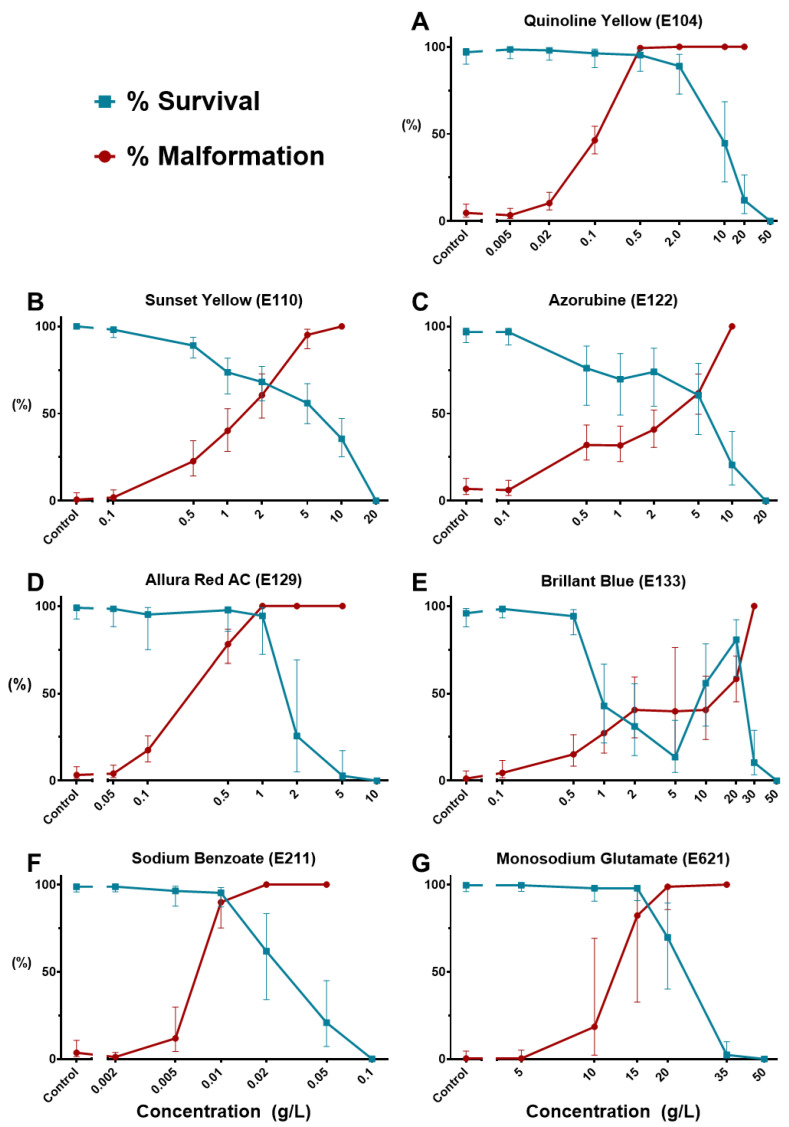
Dose–response curves showing embryonic survival (blue) and any malformation (red) upon 4-day treatment with different food additives. (**A**) Quinoline Yellow; (**B**) Sunset Yellow; (**C**) Azorubine; (**D**) Allura Red AC; (**E**) Brilliant Blue; (**F**) Sodium Benzoate; (**G**) Monosodium glutamate. Error bars showing 95% prediction intervals. Aspartame was excluded for not showing any observable effect.

Using survival and morphological (including non-hatching) data at 4 dpf, concentration–response curves were generated for the seven FAs that affected zebrafish embryonic development. As shown in Figure 2, all dose–response curves followed the typical sigmoidal pattern except those of BB (hence no LC_50_, EC_50_, or TI could be calculated). The multiphasic toxicological behavior of this colorant will be further discussed in Section 3.3.

The calculated toxicological indices at 4 dpf are listed in Table 2 (a short version of Supplementary Table S1). The table, as well as Figure 2, clearly show that the tested FAs have their toxicological indices differ by orders of magnitudes. According to our results, the preservative Sodium Benzoate (SB, E211) belongs to Cat.3 aquatic toxicity class (LC_50_~10–100 mg/L [50]), while all other compounds are non-aquatoxic with LC_50_ > 100 mg/L at 96 hpf. Although we could not determine LC_50_ values for BB and Asp, they induced no embryonic mortality at the 100 mg/L threshold, thus clearly classified as non-aquatoxic. However, all six FAs with computable LC_50_ and EC_50_ (i.e., inducing typical lethal and malformation dose–response curves) are potential teratogens with a “Teratogenic Index” (LC_50_/EC_50_) TI >1. Remarkably, the coloring agent Quinoline Yellow (QY, E104) turned out as extremely teratogenic with TI~79. It should be noted that, except for SB and teratogenicity of QY and AR, all LC_50_ and EC_50_ values are above 1000 mg/L. The NOAEC values were at 100 mg/L or lower, except for MSG and Asp (Table 2).

**Figure 3:**
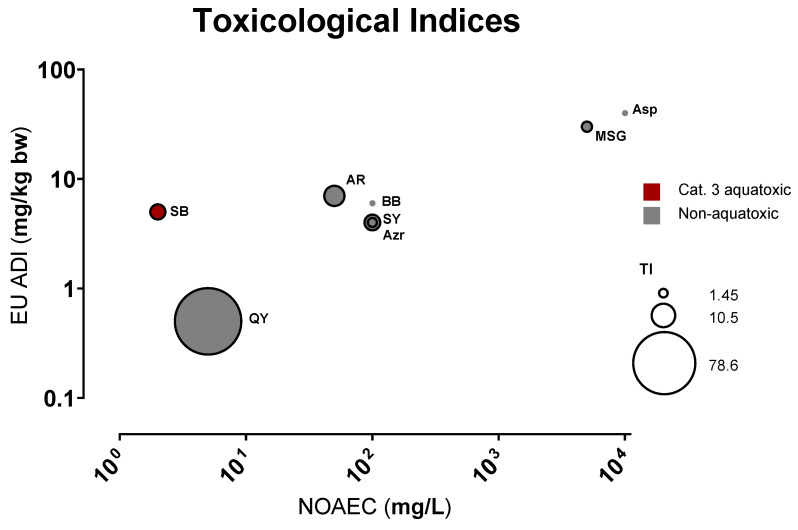
Toxicological indices of the tested FAs. NOAECs are plotted against their European ADIs [34,35,36,37,39,40,41,42]. Food additives abbreviations as listed in Table 1; red color indicates aquatoxic, grey non-aquatoxic compounds. The bubble sizes represent the teratogenic index (TI); Substances without computable LC_50_ and EC_50_ (thus no TI) are displayed as small, borderless dots.

We then compared the observed NOAEC concentrations to the “Acceptable Daily Intake” (ADI, Supplementary Table S1) values to assess their putative impact on human health. European ADI values were selected as the WHO/FAO counterpart sets no limit for Monosodium Glutamate (MSG, E621) consumption [33]. Figure 3 reveals a good correlation (Pearson’s *r*~0.98, *p*~0.00004) between legislative ADIs, which were determined mainly based on NOEAL on animal (mostly rodent) studies, and the NOAEC determined here on zebrafish embryos. One notable exception is SB, which has a much higher ADI relative to its zebrafish toxicity (see also below Section 3.4).

### 3.2. Effect of Non-Toxic Salts at High Concentration

In our study, all seven ionic FAs (QY, SY, Azr, AR, BB, SB, MSG) induced the darkened yolk phenotypes. Except for SB, the other six appeared to be non-aquatoxic (LC_50_ > 100 mg/L). Strikingly, for the six non-toxic compounds, dark yolk only became a common phenotype at the highest concentrations (500 mg/L and higher). This led us to suspect the role of ionic strength in this phenomenon. In zebrafish embryos, ionic regulation is carried out by the pronephros and the ionocytes on the skin (particularly surrounding the yolk sac)—both are formed around 2–3 dpf during the time window when hatching occurs [51,52]—i.e., when direct embryonic exposure to environmental water starts. Rider et al. [53] showed that treatment with the nephrotoxin gentamicin led to disarrayed yolk globules and darkened yolks in zebrafish embryos. A similar effect was observed upon NaCl overload (as low as 1 g/L or ~17 mM). Images of zebrafish embryos with darkened yolk could be observed in many nephrotoxicity studies, albeit rarely described or mentioned [54,55,56,57].

**Figure 4:**
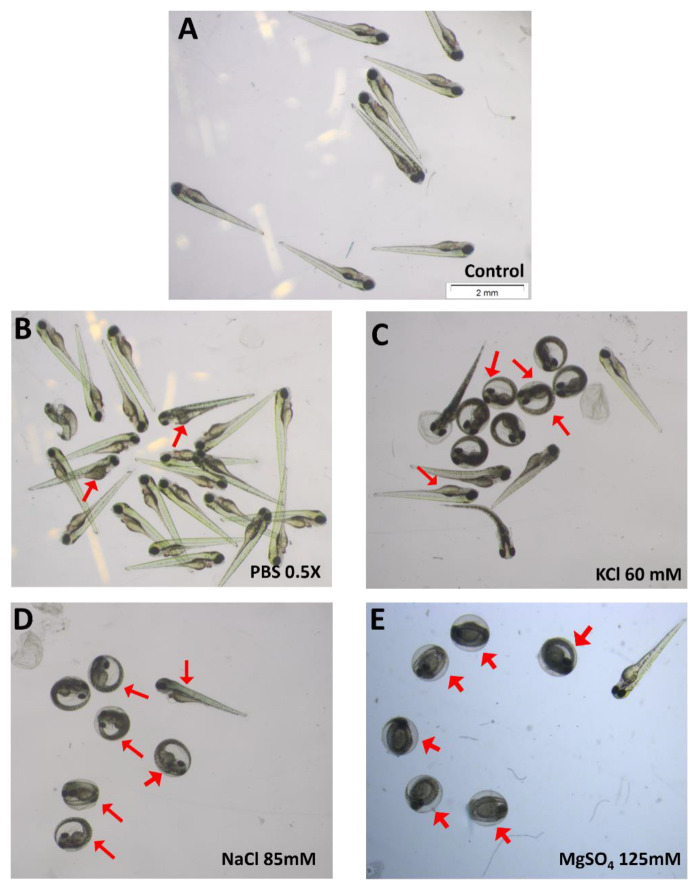
The 4 dpf embryos exposed showing different levels of darkened yolk (red arrow) in high salt solutions. (**A**) Control; (**B**) PBS 0.5X; (**C**) KCl 60 mM; (**D**) NaCl 85 mM; (**E**) MgSO_4_ 125 mM.

To investigate whether the darkened yolk phenotype was specific to the tested food additives, we performed further tests on some generally non-toxic salts such as KCl, NaCl, MgSO_4_, and PBS. Starting at 3 dpf and becoming more evident at 4 dpf, embryos treated with all these salts displayed the distinctly darkened yolk phenotype (Figure 4) at concentrations above 50 mM.

It should be noted that K^+^, Na^+^, Mg^2+^, Cl^−^, and SO_4_^2−^ are all components of the E3 medium used to raise embryos. Therefore, the most plausible explanation is that this darkened yolk is induced by the ionic strength of the solution. Interestingly, the osmolarity level that induced darkened yolk was not necessarily hypertonic to the embryos (e.g., 0.5 × PBS = 80 mM). In addition to dark yolk, Figure 4 also displays some other more general defects, such as heart edema and non-hatching.

Thus, it appears that the darkened yolk results from renal malfunction, either through high ionic strength in the medium or through nephrotoxic treatment. However, dark yolk was previously described in early zebrafish larvae as an indication for dysregulated lipid metabolism [58,59], which we cannot at present completely rule out, especially for MSG that is causing diabetes after several weeks of treatment in mice [60]. These two mechanisms may be somehow linked, the exact mechanism will require more investigations, one plausible explanation could be that failure in homeostatic regulation may lead to impaired lipoprotein biogenesis, thus causing the darkened yolk [59]. Either way, the dark yolk phenotype, while often overlooked, could serve as an indicator of possible nephrotoxicity/renal damage in other studies.

### 3.3. Brilliant Blue (E133) Can Weaken the Zebrafish Larval Yolk Sac

**Figure 5:**
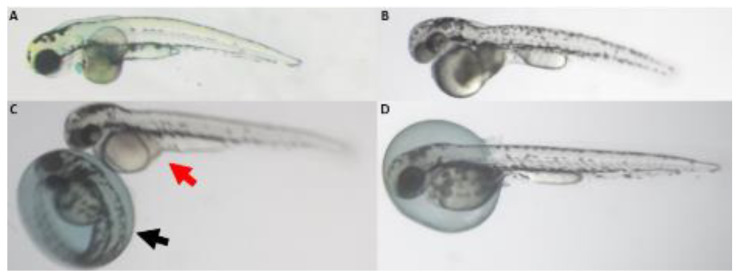
Brilliant Blue treatment weakened the zebrafish embryonic yolk sac. Hatched embryos ((**A**–**C**) red arrow) had their yolk sac ruptured, while unhatched embryos remained intact ((**C**) black arrow). During hatching (**D**), the chorion injured the softened yolk, causing its rupture and finally killing the larva. Images were taken at 2-dpf and 5 g/L BB.

As shown in Figure 2E, the coloring agent BB affected zebrafish embryos in an unusual dose-dependent manner, with the 4-day embryonic survival rate dropping between 1–5 g/L then temporarily rising again. It also caused a “ruptured” yolk sac phenotype (Figure 1E and Figure 5A,B), but that only occurred in hatched embryos which eventually died at day 4. In contrast, unhatched embryos were visually unscathed inside the chorion up to 20 g/L of BB, apparently due to a chorionic protecting effect (it should be noted that 4 dpf non-hatching was considered a defect). However, closer monitoring of the hatching process of 2–3 dpf treated larvae revealed that the chorion did not play a protective role; rather it appeared that the chorion itself was breaking and squeezing the larval yolk sac during hatching, indicating a severely softened yolk and/or weakened enveloping layer (Figure 5C,D).

Therefore, the scenario causing BB’s unusual dose–response curves is explained as follows: With increasing BB concentration up until 5 g/L, the embryonic yolk sac was weakened, but many embryos were still able to hatch, severely injuring themselves during the process leading to later death. At higher concentrations of 10 and 20 g/L, the treated embryos became unable to hatch (thus avoiding the hatching injury) while their morphology was apparently less altered. Finally, the highest BB concentrations of 30 and 50 g/L induced mortality in all unhatched embryos (Figure 2E).

While our search for yolk sac rupture during hatching in zebrafish yielded no result, there were several reports of similar phenomena in other fish species exposed to oil derivatives [61], tetrachlorodibenzo-p-dioxin (TCDD) [62,63], and silver nanoparticles [64]. However, no study suggested a mechanism of yolk sac weakening. Possible target prediction analysis revealed 36 potential protein targets (Supplementary Table S2), involving various biological processes and diseases. Experimental evidence on BB’s bioactivities also hints at several possibilities. The dye was shown to inhibit mouse oocyte pannexin 1 [65], modulating purinergic signaling and the oxidative state in skeletal muscles [66]. BB could also modulate the activity of tyrosine phosphatases (e.g., PTP1B and YPTP1) [67] and inhibit mitochondrial respiration [68]. Notably, the compound can directly penetrate animal epithelium [69] and the zebrafish chorion (Figure 1E and Figure 5C,D); hence it could possibly get inside the yolk sac and its protective walls at early periods. Taken together, these observations raise serious concern about the risk posed by BB consumption, especially during pregnancy.

### 3.4. Quinoline Yellow (E104): A Possible Metabolic Interferer

**Figure 6:**
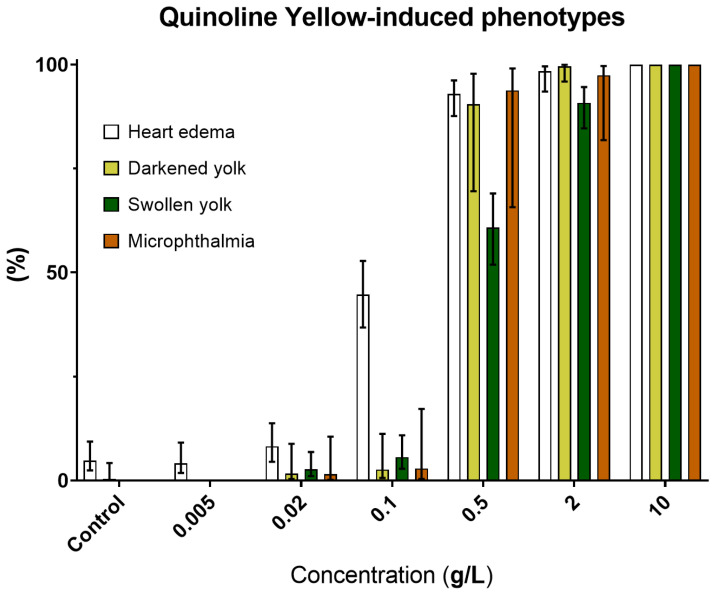
Prominent defects after four days of QY treatment to zebrafish embryos. Error bars showing 95% prediction interval.

Despite being non-aquatoxic with an LC_50_ of 6.89 g/L, the coloring agent QY was the most teratogenic food additive with an extremely high TI (~80) and lowest observed adverse effect concentration of 20 mg/L (Figure 2A). Indeed, QY is the only additive other than SB causing malformed embryos at below 100 mg/L. In addition to more general (e.g., pericardial edema) and ionic strength-induced (darkened yolk) phenotypes, QY also caused two other substance-specific deformities: microphthalmia (small eyes) and swollen yolk (Figure 1D and Figure 6).

The yolk sac, consisting of a lipid- and protein-rich core and a peripheral yolk syncytial layer (YSL), is the sole nutritive supply of the developing zebrafish embryos. The swollen yolk is likely related to the malabsorption of yolk nutrients [28,70]. Interestingly, yolk malabsorption has also been listed as a factor associated with microphthalmia [70], among other factors such as developmental delay or corneal and retinal defects [71,72]. Target prediction analysis further strengthens the hypothesis of QY impairing yolk metabolism, as 33/54 of QY hits are involved in lipid and protein metabolisms (Supplementary Table S3). It should be noted that while QY may interfere with yolk metabolism, the darkened yolk phenotype mostly occurred at very high concentration, thus more likely to be related to the ionic effect rather than a consequence of disrupted lipoprotein biogenesis as shown in other studies [58,59]. Regarding the ophthalmic effect, a recent study on the transcriptomic effect of a QY’s unsulfonated form (Quinoline Yellow SS, Solvent Yellow 33) also reported the downregulation of metabolic genes in zebrafish embryos- especially the disruption of the retinoic acid signaling pathway, which may impair eye development [22].

The implication that a common food additive may interfere with nutrient metabolisms, even at a relatively low dose, should raise a concern about its safety status at current ADIs (0.5 and 3 mg/kg bw, respectively set by EFSA and JECFA) [36,43].

### 3.5. Sodium Benzoate (E211): Safety Concern

**Figure 7:**
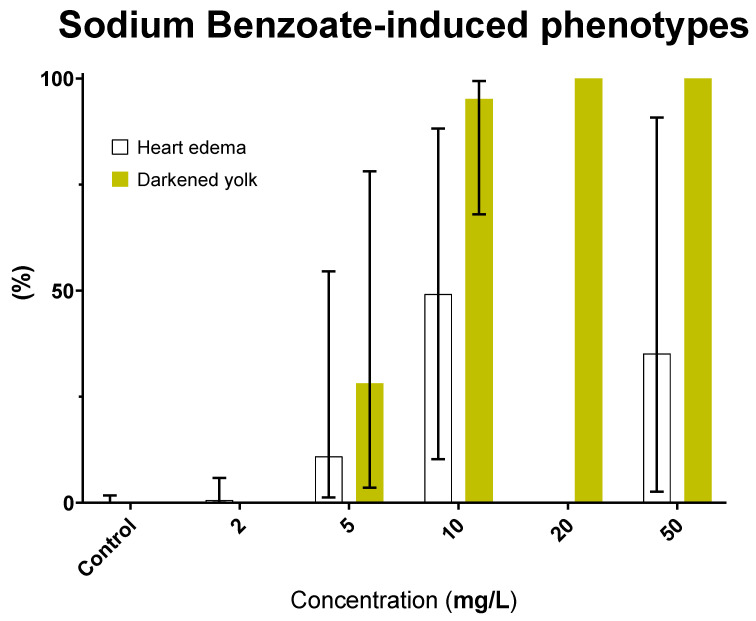
Prominent phenotypes in zebrafish larvae after four days of exposure to SB. Error bars showing 95% prediction interval.

Our results revealed SB as the most aquatoxic FA with a LC_50_ of 26.9 mg/L. Interestingly, SB also induced the darkened yolk phenotype at concentration as low as 5 mg/L (Figure 7)—much lower compared to the other FAs, thus the role of ionic strength could be eliminated. Instead, this phenomenon hinted at a specific nephrotoxic activity for SB. Indeed its consumption at 100~500 mg/kg bw/day has recently been shown to induce kidney damage in Wistar rats [73] and mice [74]. Additionally, 57/249 of SB’s potential protein targets were involved in human renal disease (Supplementary Table S4). However, although no direct link was found in the literature, 31/249 hits were related to lipid metabolism, thus we cannot rule out the lipoprotein mechanism of yolk darkening in SB-treated zebrafish embryos.

As a preservative, SB is extensively used in various food and cosmetic products. The current European ADI for SB is 5 mg/kg bw, higher than those applied for QY, SY, and Azr (Figure 3 and Supplementary Table S1). On the other hand, the JECFA (Joint FAO/WHO Expert Committee on Food Additives) has increased the ADI of benzoic acid and its salts from 5 mg/kg bw to 20 mg/kg bw in 2021 [45]—citing a NOAEL value of 1000 mg/kg bw/day obtained during an extended one-generation reproductive toxicity (EOGRT) study on Sprague-Dawley rats [75]. The evidence on SB’s status as putative nephrotoxicant, aquatoxicant, or lipoprotein disruptor emphasizes the need to reconsider its safety levels (as well as other benzoate compounds’) in food and cosmetic products, especially for those used during pregnancy and childhood.

### 3.6. Azorubine (E122) Induces Precocious Zebrafish Hatching

**Figure 8:**
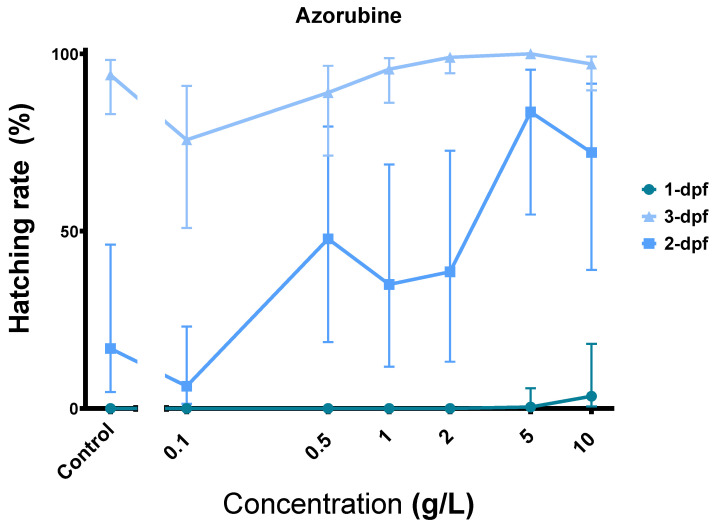
Azorubine stimulates embryo hatching even on day 1. Error bars showing 95% prediction interval.

Another remarkable observation was that Azr could act as a powerful hatching stimulant. As illustrated in Figure 8, Azr exposure dose-dependently stimulated embryonic hatching. Notably, at 10 g/L, the compound could induce some hatching at 1 dpf.

Among the tested FAs, Azr has low toxicity with a NOAEC of 100 mg/L and TI of 1.45; thus, our study does not really challenge its safety status. Nevertheless, the hatching induction feature indicates a certain biological effect of the compound. Zebrafish can hatch thanks to the combined effect of choriolytic hatching enzymes (generally belonging to the metalloprotease family) [76] and embryonic movements. Early hatching could be an adaptive response to environmental cues such as ionic stress [77] and hypoxia [78], or a consequence of chemical exposure such as tributyltin [79] and TiO_2_ [80]. While hatching stimulation could sometimes be attributed to the embryonic hyperactivity induced by chemicals, e.g., in the case of PFOS [81,82], there are compounds that induced premature hatching and reduced larval locomotion, such as tributyltin [79].

Reverse docking revealed 118 potential targets of Azr, six of which are matrix metalloproteases (MMPs 1, 2, 3, 8, 9, and 14) and ten other proteases (Supplementary Table S5). In our experiments, Azr-treated embryos did not exhibit significant hyperactivity compared to the controls. This and the fact that some embryos hatched since day 1 strongly suggest that Azr may have induced precocious hatching in zebrafish by interacting with the hatching enzymes. On the other hand, if proven, the interaction between Azr and MMPs may have practical implications in biomedicine.

## 4. Conclusions

Our results confirm the zebrafish embryo as a cost-effective model for high throughput chemical safety and toxicity screening, although specific results may need to be confirmed in more costly and more time-consuming mammalian systems. In addition, starting with routine toxicological testing using the zebrafish embryos, the careful observation of unexpected effects beyond the standard list of endpoints [19] has allowed us to uncover novel biological properties of several commonly used food additives. On the one hand, this emphasizes the advantage of whole organism in vivo models in allowing systematic detection of biological effects, especially those largely unforeseen by targeted in vitro and in silico techniques. Our deliberate effort to reach lethality for all tested compounds revealed that lethal or teratogenic doses are generally high compared to what would be reached in food or the environment. On the other hand, our results also suggest the need to reconsider the safety of QY, BB, SB, and other controversial food additives in further studies, as well as pave the way to further applications based on the newly found properties of Azr and BB.

## Figures and Tables

**Table 1:** List of tested food additives in this study.

FAs (Abbr.)	Chemical Structure	E Number	Usage	Supplier (Cat #)
Quinoline Yellow WS (QY)	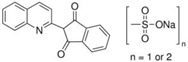	E104	Coloring agent	Sigma Aldrich (309052)
Sunset Yellow (SY)	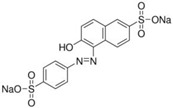	E110	Coloring agent	Sigma Aldrich (465224)
Azorubine (Azr)	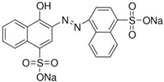	E122	Coloring agent	Sigma Aldrich (214515)
Allura Red AC (AR)	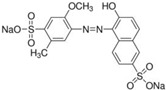	E129	Coloring agent	Sigma Aldrich (458848)
Brilliant Blue (BB)	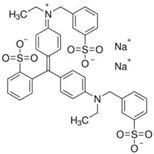	E133	Coloring agent	Sigma Aldrich (861146)
Sodium Benzoate (SB)	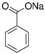	E211	Preservative	Sigma Aldrich (109169)
Monosodium Glutamate (MSG)	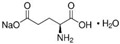	E621	Flavor enhancer	Sigma Aldrich (49621)
Aspartame (Asp)	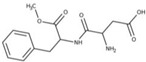	E951	Sweetener	Alfa Aesar (J61523)

Chemical structures were taken from the suppliers’ respective website Sigmaaldrich.com; alfa.com; all accessed on 15 December 2022.

**Table 2:** Toxicological indices of the tested food additives.

FA	NOAEC (mg/L)	LC_50_ Estimate (mg/L)	LC_50_ 95% CI (mg/L)	EC_50_ Estimate (mg/L)	EC_50_ 95% CI (mg/L)	TI	TI Range
QY	5	6.89 × 10^3^	5.39–8.38 × 10^3^	87.7	73.1–102	78.6	52.7–115
SY	100	5.27 × 10^3^	4.06–6.50 × 10^3^	1.20 × 10^3^	1.01–1.38 × 10^3^	4.41	2.93–6.44
Azr	100	3.97 × 10^3^	3.02–4.91 × 10^3^	2.73 × 10^3^	1.79–3.67 × 10^3^	1.45	0.82–2.74
AR	50	1.84 × 10^3^	1.57–2.11 × 10^3^	253	206–301	7.26	5.21–10.3
BB	100	N/A	N/A	N/A	N/A	N/A	N/A
SB	2	26.9	23.8–30.0	6.63	6.21–7.06	4.05	3.37–4.83
MSG	4500	20.1 × 10^3^	19.2–21.1 × 10^3^	10.9 × 10^3^	10.4–11.5 × 10^3^	1.85	1.68–2.03
Asp	10,000	N/A	N/A	N/A	N/A	N/A	N/A

CI: Confidence interval; N/A: Not applicable.

## Data Availability

All raw data are available from D.D.-T. and M.M. upon request.

## Supplementary Materials

The following supporting information can be downloaded at: https://www.mdpi.com/article/10.3390/toxics11010008/s1: Table S1: Toxicological indices of studied food additives. Tables S2–S5: SwissTarget and TargetNet results for the indicated compounds.
